# Obsessive–compulsive symptoms and brain lesions compatible with multiple sclerosis

**DOI:** 10.1007/s00702-023-02737-z

**Published:** 2024-01-30

**Authors:** Katharina von Zedtwitz, Ludger Tebartz van Elst, Horst Urbach, Sergiu Groppa, Miriam A. Schiele, Harald Prüss, Katharina Domschke, Oliver Stich, Luciana Hannibal, Dominique Endres

**Affiliations:** 1 Department of Psychiatry and Psychotherapy, Medical Center - University of Freiburg, Faculty of Medicine, University of Freiburg, Freiburg, Germany; 2 Department of Neuroradiology, Medical Center - University of Freiburg, Faculty of Medicine, University of Freiburg, Freiburg, Germany; 3 Department of Neurology, Focus Program Translational Neuroscience, Rhine-Main Neuroscience Network, University Medical Center of the Johannes Gutenberg University Mainz, Mainz, Germany; 4 Department of Neurology and Experimental Neurology, Charité—Universitätsmedizin Berlin, Berlin, Germany; 5 German Center for Neurodegenerative Diseases (DZNE), Berlin, Germany; 6 Department of Neurology and Neurophysiology, Medical Center - University of Freiburg, Faculty of Medicine, University of Freiburg, Freiburg, Germany; 7 Laboratory of Clinical Biochemistry and Metabolism, Department of General Pediatrics, Adolescent Medicine and Neonatology, Medical Center - University of Freiburg, Faculty of Medicine, University of Freiburg, Freiburg, Germany

**Keywords:** OCD, Multiple sclerosis, Inflammation, RIS, Autoimmune, CSF

## Abstract

Autoimmune-mediated obsessive–compulsive disorder (OCD) can occur in multiple sclerosis (MS). Here, a well-studied case study of a patient with OCD and MS-compatible diagnostic findings is presented. The 42-year-old female patient had displayed OCD symptoms for 6 years. Magnetic resonance imaging (MRI) identified several periventricular and one brainstem lesion suggestive of demyelination. Cerebrospinal fluid (CSF) analyses detected an increased white blood cell count, intrathecal immunoglobulin (Ig) G and IgM synthesis, CSF-specific oligoclonal bands, and a positive MRZ reaction. Neopterin was increased, but sarcoidosis was excluded. In the absence of neurological attacks and clues for MRI-based dissemination in time, a radiologically isolated syndrome, the pre-disease stage of MS, was diagnosed. Neurotransmitter measurements of CSF detected reduced serotonin levels. In the absence of visible strategic demyelinating lesions within the cortico-striato-thalamo-cortical circuits, OCD symptoms may relate to reduced intrathecal serotonin levels and mild neuroinflammatory processes. Serotonin abnormalities in MS should be studied further, as they could potentially explain the association between neuroinflammation and mental illnesses.

## Introduction

Obsessive–compulsive disorder (OCD) is a common mental disorder associated with significant impairments in various areas of life (Stein et al. 2019). OCD is assumed to have a multifactorial etiology that includes biological, psychological, and external factors (Robbins et al. 2019). At the neurostructural level, an imbalance of the cortico-striato-thalamo-cortical circuits has been suggested, while at the neurotransmitter level, serotonin alterations seem to be present (Stein et al. 2019; Robbins et al. 2019). Secondary, autoimmune-mediated obsessive–compulsive symptoms (OCS) can occur in the context of oligosymptomatic antibody-associated autoimmune encephalitis (Endres et al. 2022, 2022) and established autoimmune diseases such as multiple sclerosis (MS) (Foroughipour et al. 2012). The pathophysiological cause of the association between OCD and MS has hardly been investigated. Therefore, the rationale of this article is to present a well-studied case report of a patient with severe OCS and MS-compatible diagnostic findings in which serotonin changes may represent a pathophysiological link.

## Methods

The patient, who gave her written informed consent for this case report, received a broad diagnostic work-up based on the Freiburg Diagnostic Protocol for patients with OCD (FDP-OCD) (Runge et al. 2023). This work-up included psychometric testing as follows: the Yale–Brown Obsessive Compulsive Scale (Y-BOCS), Obsessive–Compulsive Inventory-Revised version (OCI-R), and the Structured Clinical Interview for DSM-IV (SCID-I). In addition, laboratory testing was conducted along with magnetic resonance imaging (MRI), electroencephalography (EEG), and cerebrospinal fluid (CSF) analyses with tissue-based assays. A combined volume- and region-based analysis method using the magnetization-prepared rapid gradient-echo (MPRAGE) MRI sequences and automated independent component analysis of EEG were used for automated analyses (Runge et al. 2023). Sarcoidosis parameters, an MRI of the spine, computed tomography (CT) of the chest, and electrophysiological examinations were added. In addition, the quantity of neurotransmitters and precursor metabolites in serum and CSF were measured. Quantitative profiling of neurometabolites was performed using liquid chromatography and mass spectrometry (LC–MS/MS). Plasma and CSF samples were collected on the same day, sorted for diagnostic work-up, and stored at -80^o^ C until further analysis. The methodological approach of LC–MS/MS was described in detail in an earlier publication. (Endres et al. 2022)

## Results

**Fig. 1:**
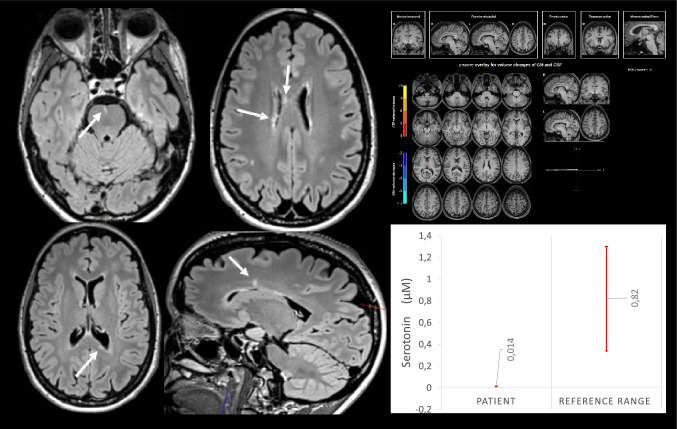
White matter magnetic resonance imaging lesions are marked with arrows. The automated magnetic resonance imaging analysis (https://www.veobrain.com/?page=veomorph) did not detect any atrophic changes. The low serotonin levels in cerebrospinal fluid are shown in comparison to known reference values from controls (Mandal et al. 2012). *CSF* cerebrospinal fluid, *GM* grey matter, *L* left, *R* right

A 42-year-old female patient demonstrated severe OCS with washing and showering compulsions for the past six years. The formal OCD diagnosis was confirmed by SCID-I. The patient’s scores on Y-BOCS and OCI-R were 28 and 30 points, respectively. The patient’s somatic history included migraine with aura. The patient had never experienced focal neurological symptoms, nor did she report experiencing fatigue. Her mother suffered from MS and OCD-like symptoms. The clinical MRI revealed eight single T2w/FLAIR patchy hyperintense lesions in the bilateral periventricular and subcortical white matter as well as one lesion in the pons. Periventricular and brainstem lesions are compatible with demyelinating MS lesions (Fig. 1). CSF analyses identified an increased white blood cell count (17 cells/microliter; reference < 5 cells/microliter), intrathecal immunoglobulin (Ig) G and IgM synthesis, as well as CSF-specific oligoclonal bands. The MRZ reaction was positive. All well-characterized antibodies against cell surface, intracellular, and glial antigens were negative (Endres et al. 2022). Tissue-based assays detected no novel central nervous system (CNS) autoantibodies. Vitamin B12 and folic acid were within the normal range, while Vitamin D was also within the normal range (31.1 ng/ml; ref. > 20.0 ng/ml), but was substituted in order to achieve highly normal vitamin D levels. Neurotransmitter measurements identified reduced serotonin concentrations of 0.014 μM in CSF (reference range 0.82 ± 0.48 μM (Mandal et al. 2012)). In addition, the sarcoidosis parameter neopterin was strikingly elevated. In the CT of the chest, no sarcoidosis-associated abnormalities were identified. Further diagnostics with electrophysiological examinations and an MRI of the spine revealed no pathologies (Table 1). In the absence of neurological attacks with focal neurological symptoms and no MRI-documented dissemination in time, radiologically isolated syndrome (RIS) was diagnosed because of inflammatory MRI and CSF changes (Thompson et al. 2018). The patient decided against immunomodulatory treatment, so watchful waiting was initiated. Guideline-based treatment for OCS with 100 mg sertraline (plasma levels of 22 ng/ml, reference: 10–50 ng/ml; higher doses up to 200 mg/day were not tolerated due restlessness, sleep disturbance, and dizziness) and inpatient cognitive behavioral therapy for approximately 10 weeks with exposure and response management resulted in only partial improvement of OCS. The patient’s Y-BOCS score was reduced to 23 (-18%), while her OCI-R score was reduced to 29 (-3%) points. An MRI follow-up after approximately six months showed no subclinical progression of the brain T2 lesion load.

**Table 1:** Full diagnostic findings

	Initial diagnostic findings
Psychometric scores	
Y-BOCS	28
OCI-R	30
Serum antibodies, immunological markers and serologies	
Anti-thyroid antibodies (against TPO, TG and TSH-receptor)	Negative
ANAs (*on HEp-2 cells*), ANCAs (*on EthOH- /formalin-fixed neutrophils*), APAs	ANAs negative, ANCA (EthOH-fixiert, IgG, 1:10): (( +)c), APAs negative
Complement factors (C3, C4)	Normal
IgG, IgM and IgA levels	Normal
CRP	< 3.0 mg/L (ref.: < 5 mg/L)
Anti-streptolysin-O	Normal
Anti-DNaseB	Normal
Rheumatoid factor	Negative
Serology for Lyme disease or lues	Negative
Paraneoplastic IgG antibodies against intracellular antigens	Negative
Well-characterized neuronal IgG cell surface antibodies	Negative
Anti-MOG/AQP4-IgG antibodies	Negative
Tissue based assay on unfixed murine brain tissue (Prof. Prüss)	Negative
Sarcoidosis parameters (IL-2-R, ACE, neopterin)	**Neopterin 31.3 nmol/L** (ref.: < 10 nm/L), IL-2-R: 328 U/ml (ref. 158–623 U/ml), ACE: 56.4 U/L (ref.: 12–82 U/L)
Serologies (CMV, EBV, HBV, HCV, HIV, tuberculosis)	**EBV positive** (Anti-EBNA1-IgG: 18.98 (ref.: < 0.8)), CMV, HBV, HCV, HIV negative
Neurotransmitters and precursors from serum*	Reduced **citrate** (25 µM; reference range: 100–150 µM), and elevated concentrations of aromatic amino acids **tryptophan **(169 µM; reference range 43–89 µM) and **phenylalanine** (150 µM; reference range 28–85 µM). All other values were normal
Cerebrospinal fluid	
White blood cell count	**17/µL** (ref.: < 5/µL)
Protein concentration	304 mg/L (ref.: < 450 mg/L)
Albumin quotient	3.1 (ref.: < 6.5)
IgG-index	**2.72** (ref.: < 0.7)
Oligoclonal bands in serum/CSF	Negative/**Positive**
Well-characterized neuronal IgG cell surface antibodies	Negative
Local IgG/IgA/IgM synthesis	**IgG synthesis 76.6%**, no IgA synthesis, **IgM synthesis 21.2%** (ref.: < 10%)
MRZ Reaction	**Positive** (ASI Measles-IgG 8.33 (ref.: < 1.5), ASI Rubella-IgG 3.7 (ref.: < 1.5), ASI VZV-IgG 3.6 (ref.: < 15)
Tissue based assay on unfixed murine brain tissue (Prof. Prüss)	Negative
Neurotransmitters and precursors from CSF*	Reduced **citrate** (25 µM; reference range 176 ± 50 µM), **succinate** (1.1 µM; reference range: 29 ± 5 µM), **glutamate** (3.5 µM; reference range: 33 ± 7 µM), **serine** (14 µM; reference range: 42 ± 15 µM), **glutamine** (113 µM; reference range: 440 ± 80 µM), **threonine** (12 µM; reference range: 28 ± 5 µM) and **serotonin** (0.014 µM; reference range 0.82 ± 0.48 µM), as well as low-normal dopamine (0.037 nM; reference range: 0.04–4.5 nM) and slightly elevated GABA (0.215 µM; reference range 0.1270 ± 0.0052 µM). 5-hydroxyinolacetic acid (5-HIAA) concentration was normal (0.103 µM; reference range: 0.055–0.163 µM). All other values were also normal
MRI of the neurocranium	
Visual inspection	Periventricular accentuated medullary lesions supratentorial on both sides with involvement of the temporal lobe, which exceeds the age limit. In total, 8 small lesions, two of which two would be **compatible with MS** (in pons and periventricular)
Automated morphometry	Normal
EEG	
Visual analyses	No intermittent/ generalized slowing, no epileptic activity
ICA	Normal
Electrophysiological investigations	
VEP	Normal
SEP	Normal
MEP	Normal
OCT	Normal
Corona (vaccination) status	Three doses of a COVID-19 vaccine, no infection

↑ means increased. *The following neurometabolites were measured in serum and CSF: Homocysteine, Cysteine, Cysteamine, Cystathionine, Methionine, Glutathione, Methionine sulfoxide, S-adenosylmethionine, S-adenosylhomocysteine, Creatinine, Argininosuccinic acid, Taurine, Hypotaurine, Homotaurine, Lanthionine, 3-Mercaptopyruvate, Dihydrofolate, 5- Methytetrahydrofolate, Tetrahydrofolate, 5,10-methylene-tetrahydrofolate, Cysteinylglycine,alpha-ketoglutarate, Citrate, Itaconate, Lactate, Malate, Malonate, Methlymalonic acid, Succinate, 2-methylcitrate, Phosphoenolpyruvate, Adenosine, Glucose, Glyceraldehyde-3-phosphate, Glycine, Alanine, Serine, Proline, Valine, Leucine/isoleucine, Aspartic Acid, Lysine, Glutamic Acid, Methionine, Histidine, Arginine, Tryptophan, Tyrosine, Asparagine, Glutamine, Phenylalanine, Threonine, Serotonin, 5-hydroxyinolacetic acid, GABA (gamma-aminobutyric acid), Dopamine, Norepinephrine, Acetylcholine, Choline, and 2-Amino adipic acid

*ACE* angiotensin converting enzyme, *ANAs* antinuclear antibodies, *ANCAs* anti-neutrophil cytoplasmic antibodies, *MOG* myelin oligodendrocyte glycoprotein, *APAs* antiphospholipid antibodies, *AQP4* aquaporin-4, *CMV* cytomegalovirus, *CRP* C-reactive protein, *CSF* cerebrospinal fluid, *EBV* Epstein-Barr virus, *EEG* electroencephalography, *HBV* hepatitis B virus, *HCV* hepatitis C virus, *ICA* independent component analysis, *IgA/G/M* immunoglobulin A/M/G, *IL-2-R* interleukin-2 receptor, *IRDA* intermittent rhythmic delta activity, *MEP* motor evoked potentials, *MRI* magnetic resonance imaging, *MRZ* antibody indices against measles, rubella, and varicella zoster virus, *OCI-R* obsessive–compulsive inventory-revised, *ref.* reference, *SEP* somatosensory evoked potentials, *TG* thyroglobulin, *TPO* thyroid peroxidase, *TSH* thyroid-stimulating hormone, *VEP* visual evoked potential, *VZV* varicella zoster virus, *WBC* white blood cell, *Y-BOCS* Yale-Brown obsessive compulsive scale

## Discussion

This paper presents a case with severe OCS and inflammatory MRI and CSF findings compatible with MS without any focal neurological symptoms (which, therefore, was formally diagnosed as RIS). In the absence of visible strategic lesions, OCS might be explained by reduced serotonin levels in the CSF. In addition, more subtle and not overtly detectable neuroinflammation might play a role in the pathophysiology here.

Relatively little is known about the relationship between OCS/OCD and MS/RIS. A PubMed search (conducted on August 27, 2023) using the search terms “(OCD OR obsessive–compulsive disorder) AND (MS OR multiple sclerosis OR encephalomyelitis disseminata OR radiologically isolated syndrome OR RIS)” identified 279 relevant papers. These publications demonstrate that the lifetime prevalence of OCD in patients with MS is at least three times higher than it is in the general population (Korostil and Feinstein 2007). The frequency of OCS in MS has been reported to be up to 31% and correlates with the severity of MS (Foroughipour et al. 2012; Khatri et al. 2021; Uguz et al. 2008). MS patients in the exacerbation phase displayed significantly higher rates of OCS than patients in the remission phase. (Uguz et al. 2008)

OCS in MS/RIS may be associated with a genetic predisposition, mild neuroinflammatory processes, strategic MS lesions and frontotemporal volume loss, or neurotransmitter alterations. Recently, a significant overlap in the genetic components (*STAT3* and *NTRK2*) of OCD and MS has been described (Sepehrinezhad et al. 2022). Mild neuroinflammatory processes (e.g., T-cell dysfunction or cytokine changes) are rarely investigated but cannot be excluded (Bechter 2020; Endres et al. 2022). The high neopterin levels in the presented patient indicate a cellular immune activation derived from T-helper cells 1 and support an inflammatory process (Murr et al. 2002). Strategic demyelinating lesions within the cortico-striato-thalamo-cortical circuits or core networks related to OCD, which could not be found in our patient, may also trigger OCS in some patients (Ellwardt et al. 2022; Shephard et al. 2021; Endres et al. 2023). In addition, evidence emerged that OCS in MS could be caused by damage in the right frontotemporal cortex (Tinelli et al. 2013). At the neurotransmitter level, the excellent efficacy of selective serotonin reuptake inhibitors suggests that serotonin deficiency or dysregulation is a central pathophysiological cause of OCD (Stein et al. 2019). In line with these considerations, our patient clearly presented reduced serotonin concentrations in the CSF. In MS, several processes are suspected to lead to low serotonin levels, including the following: (1) the differential availability of the 5-HT transporter in the CNS, (2) the reduction in total tryptophan levels, and (3) the diversion of the amino acid from its synthetic pathway (San Hernandez et al. 2020). Interestingly, recent studies have also found that an increase in serotonin levels could have immunomodulatory effects (San Hernandez et al. 2020).

Clinically, the presented patient—who has never shown focal neurological symptoms—was diagnosed with RIS, which corresponds to the pre-disease stage of MS (Thompson et al. 2018). The patient benefited only partially from guideline-based treatment with sertraline and CBT (Voderholzer et al. 2022). Immunomodulatory treatment was not desired; therefore, watchful waiting was started. Nevertheless, this case raises the question of whether immunomodulatory treatment might be associated with better outcomes in similar cases. Randomized controlled trials are needed to answer this question. Such trials could discern whether immunotherapies could provide causal treatment in similar situations or whether the presently identified association is just a comorbidity between OCD and MS/RIS.

Future research should investigate the causes of serotonin changes in MS as such studies may explain the association between MS and mental illnesses, such as OCD, depression, and fatigue.

## Data Availability

All necessary data can be found in this paper.
